# Vitamin D as a Neuroactive Substance: Review

**DOI:** 10.1100/tsw.2006.25

**Published:** 2006-01-26

**Authors:** Stephen J. Kiraly, Michael A. Kiraly, Rick D. Hawe, Naila Makhani

**Affiliations:** ^1^ Department of Psychiatry, University of British Columbia, BC, Canada; ^2^ Department of Physiology, University of Toronto, Ontario, Canada

**Keywords:** brain-derived neurotrophic factors (BDNF), calcidiol, calciferol, calcitriol, cholecalciferol, experimental autoimmune encephalitis (EAE), parathyroid hormone (PTH), vitamin D, nerve growth factor (NGF), nitric oxide

## Abstract

The objectives of this paper were (1) to review recent research on the actions of vitamin D as a steroid derivative with neuroactive properties and (2) to highlight clinical relevance and need for more research. Our methods included review of research from current journals, Medline, and Cochrane Reviews; theoretical discussion. Scientific research has had a justifiably strong emphasis on how vitamin D affects calcium metabolism and bone. This appears to have eclipsed its fundamental actions on several other important systems, including the central nervous system. Vitamin D as a neuroactive compound, a prohormone, is highly active in regulating cell differentiation, proliferation, and peroxidation in a variety of structures, including the brain. Vitamin D insufficiency is not rare. Historically, focus has been on bone metabolism, which appears to have caused *research bias* and *evidence bias*, distorting physiological importance. The central nervous system is increasingly recognized as a target organ for vitamin D via its wide-ranging hormonal effects, including the induction of proteins such as nerve growth factor. We need more research on this important neuroactive substance because it may play a role as a relatively safe and inexpensive pharmaceutical in the prevention and treatment of a number of common neuropsychiatric conditions.

## INTRODUCTION

Neurosteroids, fundamental in the nervous system, have been a veritable Pandora's box because of their large numbers, complexity, generalized distribution, and diverse physiological properties[1]. One clear aspect remains: the formation and effects of neuroactive steroids show peculiar characteristics with respect to the central (CNS) and peripheral nervous systems (PNS)[1,2]. Although vitamin D* is not a true steroid, it is a derivative of cholesterol and has been discussed in the context of its neuroactivity

* The metabolism of vit-D2 and vit-D3 are similar and some metabolites are active; therefore, in this paper, we will refer to vit-D2, vit-D3, their metabolites, and analogues, generically as vit-D.

within the CNS and PNS, as if it was a neuroactive steroid[1,2,3]. It does not qualify on the basis of chemical structure, but it certainly behaves like a neuroactive steroid and we know that neurosteroids have fundamental and far-reaching autocrine and paracrine effects in brain tissue[4]. Researchers have alluded to it as a "neglected neurosteroid" because chronic insufficiency, relatively common in many populations, has been overlooked and its role in neurodevelopmental and neurodegenerative disorders has been neglected[3,5,6]. Vit-D plays a major role in bone mineral homeostasis by enabling the transport of calcium and phosphate to ensure that the blood levels of these ions are sufficient for the normal mineralization of type-I collagen matrix in the skeleton[7,8]. Historically, physicians assumed that vit-D levels were adequate if people escaped rickets. Osteomalacia was rare and it was assumed that adults required less vit-D than children.

Demographics have changed. Given the epidemic of osteopenia and osteoporosis among swelling populations of elders, vit-D and calcium metabolism have gained priority in research and scientific inquiry. This *research bias* and the resultant *evidence bias* overshadowed the importance of vit-D as a true neuroactive compound with fundamental and far-reaching properties relevant to neuropsychiatry. Scientists have forgotten that the last biosynthetic step, the formation of the active form, 1,25-dihydroxy-vitamin-D, also occurs in the brain[1]. Vit-D receptors (VDRs), well studied in animal models, have been identified in the human brain[9].

Apart from traditional roles of calcium ion homeostasis, vit-D induces the expression of CNS genes[10,11]. Research evidences that it is one of the most potent up-regulators of nerve growth factor (NGF)[12,13,14]. For the above compelling reasons, it is timely to have a fresh look at the neuropsychiatric implications of vit-D.

## EPIDEMIOLOGY

Vit-D deficiency states are a bigger problem than originally thought. Preclinical vit-D deficiency is known in the literature as vit-D insufficiency; it would be appropriate to call it subclinical vit-D deficiency. It is common among children worldwide[15] and teens; 24% of otherwise healthy adolescents have been noted to have deficiency[16]. A large U.S. community-based survey found 9% of adult populations to be deficient (cutoff 38 nmol/l)[17]. Cross-culturally, it is surprisingly common in populations inhabiting northern latitudes[15,18,19]; 30% of healthy Canadian adults do not get enough vit-D and 25% of the adult population is considered deficient. In Canada, between the months of October and March, Canadians are unable to synthesize vit-D[20]. Deficiency is also common in industrial regions[21], the institutionalized[20,22,23], and other elderly[24,25]. Even in subtropical regions, this population is at high risk[26]. Clothing and sunscreens prevent vit-D synthesis[27,28,29]. For example, veiled Turkish women have lower levels and increased risk of osteoporosis[30,31]. Skin pigment is also a UVB blocker[32]. Obesity is associated with vit-D deficiency[33,34]. Hospitalized patients have a higher prevalence than realized[35,36]. Even the popular press has drawn attention to the facts that milk and fatty fish offer only small amounts of vit-D, and supplements do not contain enough to sustain requirements[37,38]. According to a recent position, sunlight has health benefits that may outweigh skin cancer risk[38,39,40]. Heightened awareness is necessary to ensure adequate vit-D nutrition, particularly in northern latitudes, because vit-D deficiency is easily preventable[41,42,43,44]. We have been admonished that occult deficiency is common and repletion of vit-D and suppression of PTH reduces falls and hip fractures[41,45,46,47,48]. There appears to be additional, less obvious, but serious, morbidity. Space does not permit a full review of epidemiology and suggested normal values, but a recent *executive summary* of the topic is important recommended reading[5,21] and other reviews are germane to this issue[36].

## WHAT IS VITAMIN D?

Traditionally, vit-D was known as the “antirachitic factor”. Vit-D is actually a member of a group of substances derived from the cholesterol molecule, but not having the cyclopentanoperhydrophenanthrene ring as nucleus. The early common misconception was that vit-D3 (cholecalciferol), generated in the skin, and vit-D2 (ergocalciferol), found in certain lipids and fats, were equipotent and functionally equivalent[49,50]. Recent research suggests otherwise. The two calciferols produce similar rises in serum concentrations after administration, indicating equivalent absorption. Both produced similar initial rises in serum concentrations, but vit-D continued to rise in the vit-D3–treated subjects, whereas serum vit-D fell rapidly in the vit-D2–treated subjects and was not different from baseline on the longer term[51,52]. Area under the curve (AUC) showed an even greater and significant differential. Calculated AUC indicated relative potencies for D3:D2 as being 9.5:1[51]. The activated form, vit-D2, is ergocalciferol. Both vit-D2 and vit-D3, or cholecalciferol, are found in the circulation in humans, although the amounts of each are dependent on diet and amount of skin exposure to ultraviolet-B radiation (UVB). Although often referred to as a nutrient, vit-D is a prohormone that mediates its actions via binding to VDRs, which are principally located in the nuclei of target cells including neurons and glia. Peripherally, vit-D enables calcium absorption and, to a much lesser extent, phosphorus absorption from the small intestine. Severe deficiency is characterized by inadequate mineralization of the skeleton, the cause of rickets in children, while in adults, demineralization of the skeleton results in osteomalacia leading to osteoporosis, pathological fractures, and loss of teeth[7,41,53]. Compensatory increase in the production of parathyroid hormone (PTH) promotes bone resorption. Vit-D, with PTH and calcitonin, is an essential factor in the homeostasis of systemic calcium in most vertebrate species. As a prohormone, it is also known as ‘soltriol’ because of its association with sunshine. The skin has an enormous capacity for vit-D production and supplies the body with 80–100% of requirements. Age[54,55], ambient light (latitude, time of day, season of year)[56,57], and pigmentation[58,59] dramatically affect dermal photosynthesis.

## SYNTHESIS AND ABSORPTION

Vit-D is unique among other vitamins in human nutrition because it is the only vitamin that is a conditional one. Vit-D3 is synthesized in the skin from 7-dehydrocholesterol via nonenzymatic photochemical reactions driven by UVB; a crucial, rate-limiting step. Under conditions of inadequate photosynthesis, vit-D is an *essential nutrient*. There is actually no significant natural dietary source of vit-D. Very little vit-D2 is available from plants, but more can be obtained from fortified foods (fortified milk products and irradiated plant sterols) while vit-D3 is found in fish oils. Food sources supply only about 5% of needs. Human milk is deficient[60]. It is not easy to obtain vit-D from foods. Even fortified processed cheese, which is very high in vit-D (and fat content), delivering about 5880 IU of vit-D/56.7-g serving, may be inadequate even though vit-D in fortified processed cheese is reportedly highly bioavailable and young and older adults have similar absorption (older somewhat less). Among older individuals, consuming 600 IU of vit-D daily from cheese for 2 months was insufficient to increase serum levels during limited sunlight exposure[61]. With regard to the intestinal epithelial system, the genomic effect of vit-D was elucidated years ago[62]. Vit-D, highly fat soluble, diffuses passively from the lumen of the small intestine, into enterocytes, in micelles formed from bile acids. It is distributed widely, diffuses into adipose tissue, and easily passes the blood-brain barrier.

## METABOLISM AND EXCRETION

Absorption efficiency is high; 50–80% of ingested vit-D is absorbed and secreted by enterocytes into lymphatics as chylomicrons, entering the circulation via the thoracic duct. It is transported in blood bound to a specific alpha-globulin-vit-D–binding protein. A large fraction of circulating vit-D is extracted by the hepatocytes and metabolized via the microsomal (P450) enzyme vitamin-D 25-hydroxylase to the major circulating, measurable form, vit-D3. Further hydroxylation in the proximal renal tubules yields the activated form, vit-D2. Vit-D and its metabolites are excreted primarily via the biliary and renal routes. Vit-D2, or ergocalciferol, is hydroxylated in the liver to ergocalcidiol (25-hydroxyergocalciferol or 25(OH)D2), and further hydroxylated in the kidneys to ergocalcitriol (1,25-dihydroxyergocalciferol or 1,25$(OH)_2$D2). Correspondingly, vit-D3, or cholecalciferol, is hydroxylated in the liver to cholecalcidiol (25-hydroxycholecalciferol or 25(OH)D3), and further hydroxylated in the kidneys to cholecalcitriol (1,25-dihydroxycholecalciferol or 1,25$(OH)_2$D3). Final degradation product is excreted by the kidneys[7,62].

## INFLUENCE OF AGING ON VIT-D METABOLISM

Both animal and human studies show that vit-D status declines with age[63,64,65,66,67,68] and that chronic insufficiency is associated with morbidity[54,69]. Aging is associated with (1) calcium deficiency, due to low dietary intake; (2) decreased intestinal absorption; and (3) vit-D deficiency secondary to depletion of body stores resulting from inadequate exposure to sunlight. Hepatic hydroxylation of vit-D remains normal in elderly individuals. Recent data suggest that renal hydroxylation of vit-D, previously believed to decrease with advancing age, also remains virtually normal even when creatinine clearance declines, as is very often the case in the elderly. The balance is tipped towards lower absorption and photosynthesis while degradation and elimination remain stable. The combined deficiency in calcium and vit-D stimulates the secretion of PTH, which normalizes serum calcium levels causing age-related osteoporosis[70].

## NONSKELETAL EFFECTS

Early explanations of actions, based on what was understood about calcium chemistry decades ago, focused on osteoporosis and several other conditions such as arteriosclerosis and hypertension (attributed to calcium deposition in vessel walls), amyotrophic lateral sclerosis, and senile dementia (attributed to calcium deposition in the central nervous system), and a decrease in cellular functions, supposedly because of blunting of the difference in extracellular-intracellular calcium, leading to diabetes mellitus, immune deficiency, and other disorders[71]. Later studies suggested that vit-D is the necessary factor for a number of effects formerly attributed only to calcium. The large number of effects on a variety of tissues is better explained by vit-D’s fundamental regulatory activity acting on widespread, at least 150, specific nuclear receptors regulating cellular differentiation and proliferation[7].

## ACTIONS WITHIN BRAIN

The presence of VDRs in the brain suggested that vit-D had functions in this organ and accumulating data have provided evidence that this is indeed the case. The nuclear receptors for vit-D3 have been localized in neurons and glial cells. Genes encoding the enzymes involved in the metabolism of this hormone are also expressed in brain cells. Reported biological effects in the nervous system include the biosynthesis of neurotrophic factors and at least one enzyme involved in neurotransmitter synthesis[72]. Vit-D3 metabolites can also inhibit the synthesis of inducible nitric oxide synthase (iNOS) and increase glutathione levels, suggesting a role for the hormone in brain detoxification pathways. Vit-D is involved in the glutathione cycle and in cysteine transport. Brain gamma-glutamyl transpeptidase (gamma-GT) is increased after vit-D administration and this positive effect was noted in pericytes and parenchymal astrocytes. Gamma-GT is thought to participate in the scavenging of reactive oxygen species and it was hypothesized that vit-D could be an effector controlling detoxification processes in the brain[73].

Astrocytes play a pivotal role in CNS detoxification pathways, where glutathione is involved in the elimination of oxygen- and nitrogen-reactive species such as nitric oxide. Gamma-GT, an enzyme of central significance in glutathione metabolism, is regulated *in vivo* in astrocytes by vit-D3. It appears that both gamma-GT gene expression and specific activity, induced by lipopolysaccharide, are potentiated by vit-D3. In contrast, vit-D3 does not regulate the expression of other enzymes involved in astrocytic detoxification processes, such as superoxide dismutase or glutathione peroxidase. In parallel, vit-D3 enhanced intracellular glutathione pools and significantly reduced nitrite production induced by lipopolysaccharide. These findings suggest that gamma-GT, glutathione, and vit-D3 play a fundamental role in astrocyte detoxification pathways, which, at least in part, explain neuroprotective effects[74].

## EFFECTS ON IMMUNE SYSTEM

In humans, vit-D regulates immune function[75]. It influences the production of lymphocytes and the cytokine cascade, preventing an overproduction[76]. By suppressing overactivity of T-helper cell mobilization, which may attack a body's own cells, vit-D plays an important role in the prevention of autoimmune diseases such as type-1 diabetes, lupus, rheumatoid arthritis, scleroderma, and graft rejection[75,76,77,78]. Thus, it has been found that dark skinned (African American) women living in northern climes are at higher risk for autoimmune disease than their southern counterparts [76,79,80]. What about autoimmune disorders in brain? We know that excessive production of nitric oxide is associated with CNS inflammatory disease. The iNOS generates nitric oxide. During experimental allergic encephalomyelitis (EAE) (of the Lewis rat), there was an elevated iNOS immunoreactivity and mRNA both during inflammatory bursts and during the remission phase. iNOS expression was region specific and expanded with time along a caudo-rostral axis correlating with the development of inflammatory infiltrates. Whereas cells of the monocyte/macrophage lineage continuously contributed to iNOS expression, astrocytes only expressed iNOS immunoreactivity or mRNA during the relapse. In order to investigate possible regulatory effects of vit-D3 on iNOS expression, rats were treated with the hormone after the beginning of clinical signs and areas of the CNS were later examined. Vit-D3 exerted a drastic inhibitory effect on iNOS expression, both at the protein and the mRNA levels. iNOS down-regulation occurred in macrophages, activated microglia, and astrocytes. The inhibition of iNOS expression in some CNS structures could account for the improvement of clinical signs observed in EAE-rats treated with vit-D3. Since vit-D3 can be synthesized by activated macrophages or microglia, research results support the hypothesis that this hormone might be implicated in the control of the CNS-specific immune responses. Vit-D3 or its analogues could, thus, be of therapeutic value in the management of iNOS-associated diseases of the CNS[81]. Other research studies on EAE, used as a rodent MS paradigm, have shown that treatment of EAE by vit-D3 leads to a clinical improvement accompanied by an inhibition of nitric oxide synthase (NOS) expression in the rat brain. It has also been demonstrated that vit-D deprivation leads to moderate exacerbation of the clinical symptoms. Interestingly, curative treatment of vit-D–deprived rats with a nontoxic-1,25-D3 analogue (MC1288) strongly inhibited EAE symptoms, thus emphasizing the potential interest of such compounds in the management of multiple sclerosis[82].

## RELEVANCE TO NEUROPSYCHIATRY

Given its fundamental gene-modulating and enzyme-inducing properties, it is not surprising that vit-D has CNS and PNS effects that influence cognitive function and neuropathology. For example, a rare autosomal-dominant inheritance of idiopathic strio-pallidodentate calcifications and late onset of extrapyramidal symptoms was characterized by Parkinsonism in one member and postural tremor in two[83]. Depression and dysarthria were present in all cases. All symptomatic members showed a peculiar biochemical abnormality of vit-D metabolites suggesting an inborn error of metabolism. The biochemical, clinical, and genetic pattern of this family distinguished their syndrome from the larger group of secondary familial basal ganglia calcifications. While rare conditions cannot be ignored, common conditions attract more widespread clinical enthusiasm.

## MOOD DISORDERS

Past or current depression in women is associated with decreased bone mineral density[84]. A German study showed that vit-D levels were lower in patients suffering from schizophrenia, alcoholism, and major depression compared to healthy controls. Vit-D levels were similar in all three groups suffering from psychiatric illnesses. These findings disputed the idea that vit-D is involved specifically in the pathogenesis of major depression. The authors suggest that low levels of vit-D may be more reflective of the social and environmental circumstances of these psychiatric patients[85]. The effect of vit-D levels on major depression is not clear. Hypercalcemia, whether due to undiagnosed high PTH or some other cause, can lead to clinical depression. One patient described developed a severe depressive illness associated with hypervitaminosis-D after 15 years of inappropriate therapy with calcium gluconate and excessive vit-D3 treatment. The importance of monitoring serum calcium levels in anyone on vit-D therapy is stressed[86].

Of particular interest is “winter depression”, or seasonal affective disorder (SAD). Early research (1989) noted that effects of sunlight, or equivalent artificial light, on physiological and behavioral processes were probably mediated, in large part, through the skin-vit-D-endocrine system[87]. Experimental evidence revealed sites of action and concomitant direct effects of vit-D3 on brain, spinal cord, pituitary, and other target tissues. It was postulated that this had relevance for the activation and modulation of cerebral and endocrine processes, particularly related to seasonal and daily biorhythms, independently of calcium levels. Direct VDR actions were mediated in a dose-related manner according to intensity of light and length of exposure; in this sense, light (photons), behaved as a drug[87]. Serotonin synthesis is hypothesized to be dependent on the duration of light exposure during the previous summer[88]. Increased levels of anxiety and depression during winter occur in normal populations and are most likely related to short days with lower light levels. Some individuals are very sensitive to this decrease in sunlight and thus develop SAD. More than 80% of SAD sufferers are women with an age of onset generally in the third decade. Symptoms of SAD include carbohydrate craving, hypersomnia, lethargy, and changes in circadian rhythms. A randomized controlled trial (RCT) has shown that vit-D3 supplementation significantly enhanced positive affect and in some cases reduced negative affect in healthy subjects during winter[89]. SAD is prevalent when vit-D stores are typically low. Broad-spectrum light therapy will include wavelengths between 280–320 nm that allow the skin to produce vit-D. A prospective RCT showed that vit-D therapy was more efficacious than phototherapy[90]. There are studies that do not show this correlation[91,92].

## SCHIZOPHRENIA

Epidemiological evidence suggests that low maternal vit-D is a risk factor for schizophrenia. The CNS was increasingly recognized as a target organ for vit-D via its wide-ranging neuroactive effects and via the induction of various proteins such as brain-derived neurotrophic factors (BDNF)/NGF, and this led to the hypothesis that low maternal vit-D impacts adversely on the fetal brain, leaving offspring at increased risk of adult-onset schizophrenia. The hypothesis can parsimoniously explain diverse epidemiological features of schizophrenia, such as the excess of winter births, increased rates of schizophrenia in dark-skinned migrants to cold climates, the increased rate of schizophrenia births in urban vs. rural settings, and the association between prenatal famine and schizophrenia. Studies that will allow rejection of the hypothesis are proposed[93]. Subsequently, not all epidemiological studies confirmed the vit-D hypothesis[94,95], but evidence continued to mount. It was noted that maternal vit-D did not operate as a continuously graded risk factor. The results in the dark-skinned subgroup raised the possibility that below a critical threshold, low levels of maternal vit-D were associated with an increased risk of schizophrenia[96]. Vit-D supplementation during the first year of life is associated with a reduced risk of schizophrenia in males[97]. Preventing hypovitaminosis-D during early life appears to reduce the incidence of schizophrenia[97]. Vit-D is an attractive “environmental candidate” because it explains several key epidemiological features of schizophrenia. It is also an attractive “genetic candidate” because its nuclear VDR regulates gene expression and nervous system development. The polygenic quality of schizophrenia, with linkage to many genes of small effect, maybe brought together via this “vitamin D hypothesis”. For a discussion of the possibility of a broader set of environmental and genetic factors interacting via VDRs to affect the development of the brain leading to schizophrenia, refer to Mackay-Sim et al.[98]. The same researchers have found that rats born to vit-D3–deficient mothers had characteristic alterations in brain architecture. The cortex was longer, but not wider, the lateral ventricles were enlarged, the cortex was proportionally thinner, and there was more cell proliferation throughout the brain. There were reductions in brain content of NGF and glial cell line–derived neurotrophic factor and reduced expression of p75(NTR), the low-affinity neurotrophin receptor. These anatomical findings (not unlike the neuroimaging studies of brains of schizophrenics) would suggest that low maternal vit-D has important ramifications for the developing brain[99].

## MULTIPLE SCLEROSIS

There is correlational evidence between frequency of multiple sclerosis (MS) lesions, seasonality[100,101], and geographic latitude[102,103]. The most striking clue to the role of environment in MS has always been the latitude gradient. This is most obvious in Australia, where the risk in temperate Tasmania is fivefold that in sunny Queensland[104,105], but where ethnic origins are relatively uniform. The gradient is also seen in several other countries, including New Zealand[106], the U.S.[107], and Canada[108]. Studies of migrants indicate that risk for MS is strongly associated with place of residence early in life[105,109]. In Canada, the risk for a dizygotic twin of someone with MS is almost twice that for a full nontwin sibling[108]. This may implicate environmental factor(s) such as shared timing of gestation or birth, or both. Furthermore, the study of half siblings has shown a maternal effect that could be environmental in nature[104]. Studies of month of birth and risk of MS have been carried out in several cohorts of people with MS, but sample sizes, ethnic groups, and statistical methods differed for each study and findings have been inconsistent[110,111,112,113,114]. Month of birth and risk of MS are clearly associated, more so in familial cases, implying interactions between genes and environment that are related to latitude. Such interactions may act during gestation or shortly after birth in individuals born in the northern countries studied[115]. Given the seeming correlation with the vit-D level gradient, a protective effect of vit-D on risk of MS has been proposed. The first large prospective study confirmed the protective effect of vit-D, largely from multivitamins, against the risk of developing MS[116]. Benefits from any particular diet in MS have not been shown. Malnutrition may potentially exacerbate the symptoms of MS. Intake of vit-D is associated with a lower incidence of MS. In MS, the risk of osteoporosis is high, and prophylactic vit-D has been recommended at early stages[117]. It appears that vit-D levels, whether enhanced by diet or sunlight, contribute to decrease in MS lesion activity[26,100]. As noted, animal studies, using EAE models of MS, emphasize the idea that vit-D3 can regulate some aspects of EAE. Vit-D3 exerts regional effects on constituent cells in the CNS and can have immunomodulatory effects inside the CNS during an ongoing immune process[118]. Vit-D–related compounds remain of potential interest in the management of MS[82].

## SENSORINEURAL HEARING LOSS

Sensorineural hearing loss has been reported in vit-D–resistant osteomalacia[119] and in patients with renal failure, but its etiology has not yet been established. Researchers' findings in rats support the hypothesis that hearing loss in vit-D deficiency is mainly attributable to the depression of the calcium ion concentration in perilymph[120].

## ANTITUMOR ACTIVITY IN BRAIN

More recent works show that the VDR is expressed in both neurons and glial cells; it induces NGF and it is a potent inhibitor of mitosis and promoter of differentiation in numerous cells. Using cultured embryonic hippocampal cells and explants, researchers found that the addition of vit-D3 significantly decreases the percentage of cultured hippocampal cells undergoing mitosis in conjunction with increases in both neurite outgrowth and NGF production[121]. Vit-D3 induces glioma[72,122] and neuroblastoma[123] cell death through alterations in the expression of NGF, making the hormone of potential interest in the management of brain tumors[72].

## PARATHYROID HORMONE AND CALCITONIN

Hyperparathyroidism is the third most common endocrine disorder, increasing with age, affecting mostly postmenopausal women[7,14,124]. PTH fluctuates seasonally[92]. PTH abnormalities are associated with an impressive list of psychiatric symptoms referable to ionized calcium fluctuations and possibly to central effects of PTH itself acting on specific PTH receptors[7,125]. Elevated PTH can present as depression, fatigue, or disorientation, and this appears to be associated with poor calcium absorption[16]. PTH levels increase well before the vit-D3 level decreases to the lower limit of normal for some assays[126,127]. Thus, levels of vit-D may need to be higher than the traditional normal values to protect against secondary hyperparathyroidism[45,128,129]. Pain perception may be influenced because the transcription of the calcitonin gene is down-regulated by vit-D[130].

## HYPOTHESES REGARDING THE ROLE OF VIT-D

### Neuroprotection and Neurogenesis

VDRs are nuclear receptors that mediate the effect of vit-D. *In vitro* studies show a strong stimulatory effect of vit-D on mRNA and NGF gene expression in a variety of brain cell lines[13,123,131,132,133,134,135] and in rodent models[12,14,136]. Concentrations as low as $10^{-10}$ *M* stimulate VDR gene expression and strongly increase the numbers of activated VDRs capable of binding to specific vit-D–responsive elements (VDRE). Decreased NGF synthesis in the hippocampus and reduced NGF-receptor immunoreactivity in basal forebrain areas have been implicated in neurodegeneration, and VDRs have been located in brain areas affected by neurodegenerative diseases. Thus, vit-D3 was found to be a potent inducer of BDNF expression and it contributes to the regulation of BDNF *in vivo*[134,135]. NGF has the ability to increase cholinergic function in animal models of cholinergic brain degeneration; therefore, these results are supportive of a role for NGF inducers as potential drugs for neurodegenerative disorders[12]. Vit-D3 also enhances glial cell–derived nerve factor (GDNF) mRNA *in vitro* and *in vivo*. Rats pretreated with vit-D3 had elevated (up-regulated) cortical GDNF and showed attenuated ischemic cortical injury after middle cerebral artery ligation[137]. Exogenous administration of GDNF reduced ventral mesencephalic dopaminergic neuron damage and resultant hypokinesia, induced by 6-hydroxydopamine (6-OHDA) lesioning in rats. Neuroprotection against damages by free radical and reactive oxygen species was the purported mechanism[136]. Other studies have linked vit-D with general neuroprotection[138,139] and it has been noted to retard biomarkers associated with hippocampal degradation in rat models of Alzheimer's disease[140]. Subsequently, it was reported that vit-D3 has direct anti-inflammatory effects on microglia[141]. On the basis of NGF's ability to increase cholinergic function in animal models of cholinergic degeneration, these results are supportive of a role for NGF inducers as potential drugs for neurodegenerative disorders[12]. Such anabolic activity may be counteracted by glucocorticoids[141]. Dexamethasone is known to decrease the pool of NGF mRNA in various experimental systems. Research provides evidence that the negative regulation exerted by dexamethasone may be counteracted in a dose-dependent manner by the positive action of vit-D, and vice versa. Therefore, the expression of the NGF gene can be regulated in a subtle way by the balance between the two steroids. It may be expected on the basis of these observations that in tissues that are responsive to both hormones, administration of vit-D should be able to reverse the down-regulation of NGF synthesis elicited by glucocorticoids[141].

### The Dual Action of Vit-D in the Immune and Nervous Systems

VDRs are nuclear receptors that influence cell differentiation in many, and possibly all, tissues; therefore, it is not surprising that vit-D has manifold properties, including actions in the immune system. As noted in previous discussion, the immunomodulatory actions of vit-D have high relevance for brain tissue. This may have implications for clinical entities such as MS and Alzheimer's disease. Immunomodulatory effects of this hormone, described in several experimental models, indicate the potential value of vit-D3 pharmacological analogues not only in neurodegenerative, but also in neuroimmune, diseases[72].

## CONSEQUENCES OF VIT-D DEFICIENCY

Given that toxicity can occur, there remains considerable controversy around the level that constitutes vit-D deficiency. It has been suggested that levels at accepted norms may be inadequate to maintain biochemical normality even if manifestations of osteomalacia or hypocalcemia do not occur[6]. The serum concentration of vit-D above deficiency levels, but below what is needed to provide optimal health, has been termed vit-D insufficiency[142]. Is this not subclinical vit-D deficiency? Animal research and population studies support the notion that chronic vit-D insufficiency is harmful to brain development and optimal adult neuronal tissue maintenance. We know that, in rodent models, vit-D deficiency causes brain malformation. In human studies, vit-D insufficiency is associated with mood disorders, MS, schizophrenia, and brain tumors. Some of these conditions have been noted to respond to vit-D therapy. This possibly pandemic condition may very well constitute an identifiable prodrome worthy of early intervention because of downstream morbidity[5,143,144].

## SUMMARY AND CONCLUSION

Vit-D influences neuronal function by binding to intracellular receptors that can act as transcription factors and regulate gene expression. Fundamental effects are on cell differentiation, growth, antioxidative, and autoimmune inflammatory mechanisms. Biological effects in the nervous system appear to arise from stimulation of neurotrophic factors, quenching oxidative hyperactivity and regulating autoimmune responses.

Based on epidemiology, and on *in vitro* and *in vivo* animal experiments, vit-D has been linked to MS, schizophrenia, mood disorders, sensorineural effects, and certain brain cancers. Fetal environmental management is becoming important because pre- and perinatal vit-D levels may imprint on the functional characteristics of various tissues, leaving the affected individual at increased risk of developing adultonset disorders. This hypothesis draws from recent advances in the understanding of the early origin of adult disease and proposes a "critical window" during which vit-D levels may have a persisting impact on adult health outcomes. Methods to test the hypothesis have been outlined[97]. If correct, the hypothesis has important implications for public health. Careful attention to maternal vit-D status could translate into diverse improvements in health outcomes for the following generation[96]. Thus, high-risk groups should be monitored, not only for the emergence of skeletal disorders (rickets, osteomalacia, osteoporosis, fractures), but also for neuroimmune and neuropsychiatric effects, which may very well precede the skeletal effects. Prevention of some of these disorders may prove to be much easier than treatment[145].

Other clinical issues pertain to misdiagnosis. Iatrogenesis is possible, as chronic drug use may block vit-D. For example, the antimycotic agent ketoconazole is known to inhibit several cytochrome P450–dependent enzymes involved in the biosynthesis of steroid hormones from cholesterol. Since vit-D is also a sterol synthesized by cytochrome P450–dependent enzymes, inhibition may lead to low vit-D levels[146].

On the other hand, there may be novel therapeutic potential. Dexamethasone is known to decrease the pool of NGF mRNA in various experimental systems. Measurements of NGF mRNA and assays of the mature protein secreted by the cells provide evidence that the negative regulation exerted by dexamethasone may be counteracted in a dose-dependent manner by the positive action of vit-D, and vice versa. Therefore, the expression of the NGF gene can be regulated in a subtle way by the balance between the two compounds. It may be expected, on the basis of these observations, that in tissues that are responsive to both hormones, administration of vit-D should be able to reverse down-regulation of NGF synthesis elicited by stress hormones (glucocorticoids)[141]. Given that vit-D3 and analogues with minimal calcemic effects induce NGF synthesis *in vivo*, and *in vitro*, increasing NGF in the CNS or PNS remains a rational pharmacological strategy for treating certain neurodegenerative disorders[147]. Such observations suggested the use of vit-D to treat peripheral neuropathy (pain) by inducing NGF production in the human skin[148].

There is a paucity of prospective controlled trials regarding the effects of vit-D, but accumulating data are converging to provide evidence that vit-D is involved in brain function and this is likely to have clinical implications. The nuclear receptor for vit-D has been localized in neurons and glial cells. Genes encoding the enzymes involved in the metabolism of this hormone are also expressed in brain cells. In addition, it induces glioma cell death, making the hormone of potential interest in the management of brain tumors. These results reveal previously unsuspected roles for vit-D in brain function and suggest possible areas of future research[72]. Importantly, studies suggest that the powerful NGF-inducing effects of vit-D have a potential for the pharmacotherapy of neurodegenerative disorders[12,14,122,131,132,135, 136,137,149]. There may be an unrealized neuropsychopharmacological potential that arises from the intracellular cross-talk between genomic and nongenomic effects. Recent research reveals previously unsuspected roles for vit-D in neuropsychiatry and suggests possible areas of future exploration. Thus, chronic hypovitaminosis-D should be examined in more detail as a candidate for modifying risk factors for neurodevelopmental, psychiatric, and neurodegenerative disorders.
